# Integrating artificial intelligence into venous thromboembolism care: Predictive models, implementation challenges, and future directions

**DOI:** 10.1016/j.jvsv.2026.102608

**Published:** 2026-08-31

**Authors:** Oscar Moreno, Ruoliu Zhang, Andrew Huang, Yanqing Zhao, Amber Clay, Jorge H. Ulloa, Brajesh K. Lal, Thomas Wakefield, Minhaj S. Khaja, Andrea T. Obi, Peter Henke

**Affiliations:** a Section of Vascular Surgery, Department of Surgery, University of Michigan, Ann Arbor, MI; b Department of Biomedical Engineering, University of Michigan, Ann Arbor, MI; c Division of Vascular and Interventional Radiology, Department of Radiology, University of Michigan, Ann Arbor, MI; d Facultad de Medicina, Universidad de los Andes, Bogotá D.C., Colombia; e Department of Vascular Surgery, University of Maryland, Baltimore, MD

**Keywords:** Artificial intelligence, Venous thromboembolism, Machine learning, Risk of bias, External validation, Agentic AI

## Abstract

Artificial intelligence (AI) has the potential to support personalized, multidisciplinary, data-driven care for venous thromboembolism prevention, detection, imaging, management, and follow-up. We reviewed 23 studies; 20 prediction or detection studies were assessed with PROBAST+AI, while 3 evaluated clinical-impact interventions. Of these, 18 of 20 studies (90.0%) were at high risk of bias. Only 7 studies (35%) showed meaningful external or prospective validation, although 5 of 7 remained at high risk of bias. Calibration was not reported in 14 studies (70.0%). Discrimination scores ranged from modest to high, but performance varied across cohorts and was often limited by the retrospective design, internal validation, class imbalance, low event counts, and overfitting. Currently, agentic AI remains conceptual and needs human oversight. Future research should emphasize prospective multicenter evaluation, calibration, transparent reporting, regulatory adherence, and standardized bias auditing before AI is used routinely in clinical practice.

The current risk assessment models for venous thromboembolism (VTE) (Caprini, Padua, and IMPROVE) have improved VTE prophylaxis rates and raised awareness of VTE. However, they achieve only modest discrimination (area under the receiver operating characteristic curve [AUC], 0.56-0.60),^1^, ^2^, ^3^, ^4^ with inconsistent validation across centers.^5^^,^^6^ Artificial intelligence (AI) offers potential for task-specific personalized, dynamic risk prediction by integrating electronic medical records (EMRs), imaging, and laboratory data.^7^^,^^8^ However, the methodological robustness remains uncertain. This article assesses the risk of bias (ROB) in AI, summarizes the barriers to its clinical implementation, and offers tools to address these limitations.

## Methods

We searched PubMed/MEDLINE, Embase, and Scopus (2014-2026) for peer-reviewed AI studies on VTE care. The inclusion criteria were as follows: original research with performance metrics. The exclusion criteria were as follows: abstracts without full text and nonhuman studies. We extracted the study design, sample size, AI methodology, validation strategy, and performance metrics. ROB was assessed with PROBAST+AI across four domains: Participants, Predictors, Outcome, and Analysis, covering data sources, definitions, blinding, sample size, missing data, overfitting, performance reporting, and validation.^9^^,^^10^ A domain was considered high risk if the response to any question was No (N) or Probably No (PN). The overall risk was high if any domain was at high risk.

## Results

We have organized the leading AI applications in five venous disease areas and have summarized their methods, validation strategies, and potential clinical relevance in Table.

**Table:** Comparison of artificial intelligence models across venous thromboembolism care domains

Venous disease areas	Clinical application and AI approach	Population/validation	Key performance and comparison	Reference
VTE risk prediction	VTE diagnosis and 1-year risk prediction. EMR-based AI algorithm	159,001 Mount Sinai patients for development and internal evaluation; external cohorts: 401,723 UK Biobank and 123,039 All of Us participants (683,763 total)	Large-model AUROC for diagnosis: 0.83 internally, and 0.80 (UK Biobank) and 0.82 (All of Us) externally; 1-year risk AUROC = 0.78 internally and 0.65 and 0.69 externally; demonstrated higher discrimination than modified Padua score in the evaluated cohorts	Chen et al, 2024^11^
	VTE prediction in Chinese patients. Random forest, best of 8 ML algorithms	1955 patients: 376 in the balanced derivation cohort and 1579 in a consecutive clinical validation cohort	Random forest AUC = 0.856 ± 0.049 internally and 0.782 in clinical validation; sensitivity = 0.817 and specificity = 0.80 in derivation testing	Wang et al, 2020^12^
	Postoperative VTE prediction. MySurgeryRisk random forest using 135 variables	58,236 patients undergoing 74,417 procedures: 52,117 development procedures and 22,300 prospective validation procedures	Prospective temporal validation was performed; VTE-specific AUROC was not reported separately in the extracted results; the application displayed individualized risk and contributing factors	Ren et al, 2022^13^
	Pulmonary embolism prediction in hospitalized patients. XGBoost	331,268 admissions from 12 development hospitals; 1,660,715 admissions from 32 external hospitals (1,991,983 total admissions)	AUROC = 0.87 in internal holdout testing and mean of 0.88 across external hospitals (range, 0.79-0.93)	Shen et al, 2022^14^
	VTE prediction in ICU patients. Random forest	109,044 ICU patients from the eICU database: 72,742 training and 36,302 for internal validations	Highest validation AUC = 0.9492 with XGBoost; random forest AUC = 0.9378; no independent external validation^a^	Guan et al, 2023^15^
	Early mortality in ICU patients with VTE with or without cancer. AutoML/JADBio	2468 MIMIC-III patients with VTE identified for development; 2659 eICU patients in the external validation of the early-mortality model	Early-mortality AUROC = 0.93 internally and 0.87 externally; outcome was mortality among patients with VTE rather than incident VTE	Danilatou et al, 2022^16^
	Cancer-associated VTE prediction. ML models incorporating biomarkers including D-dimer	1035 patients with cancer: 725 training and 310 internal testing	AUC = 0.773 for linear discriminant analysis with D-dimer (logistic regression, 0.772); no external validation	Jin et al, 2022^17^
	VTE prediction in acutely ill medical patients. Super learner ensemble (39 algorithms; 68-variable and 16-variable versions)	6459 patients analyzed from the APEX trial cohort (7513 enrolled)	Cross-validated C-statistic = 0.69 for the full ML model and 0.68 for the reduced model vs 0.59 for IMPROVE	Nafee et al, 2020^18^
Detecting VTE in Unstructured EMR Data	VTE identification from unstructured clinical text. NLP vs ICD-10 codes	6049 admissions for algorithm development and a separate 4000-admission validation sample across 5 Canadian hospitals	Algorithm-specific AUCs; validation AUC = 0.96 for CT-based PE and ultrasound-based DVT detection, with high sensitivity and specificity	Verma et al, 2022^19^
	VTE confirmation from radiology reports. ClotCatcher NLP with PEGASUS-based data augmentation	2273 imaging studies from 2 hospitals: 1358 at EUH and 915 at GMH	Best reported AUC = 0.980 at EUH and 0.995 at GMH; evaluations used site holdouts from the two source hospitals rather than fully independent external validation	Wang et al, 2023 (ClotCatcher)^20^
	VTE identification from EMR data. NLP	400 manually reviewed charts: 100 PE-positive, 100 PE-negative, 100 DVT-positive, and 100 DVT-negative	Sensitivity = 93.3%, specificity = 99.6%, NPV = 99%, and PPV 97.1%; AUC was not reported	Woller et al, 2021^21^
	Incidental PE detection in unreported cancer cases. AidocBriefCase CNN	1069 patients comprising 1892 CT studies; 75 studies were positive for incidental PE	Detected 68 of 75 incidental PE cases; sensitivity = 90.7% and specificity = 99.8%; AUC was not reported	Wiklund et al, 2023^22^
	PE, DVT, and incidental-finding identification in radiology reports. NLP	573 French radiology reports	F1 scores of 0.98 for PE, 1.00 for DVT, and 0.80 for clinically significant incidental findings; AUC was not reported	Pham et al, 2014^23^
Clinical decision support and Intervention outcome prediction	Hospital-associated VTE prevention. AI-CDSS integrating structured and unstructured EMR data and recalculating Caprini, Padua, bleeding, and Wells scores	Prospective randomized controlled trial; 19,785 hospitalized patients	46% reduction in hospital-associated VTE (absolute risk reduction: 0.23; control; 0.5, intervention: 0.27); 24% increase in mechanical prophylaxis; 9.72% increase in intensity drug use.	Huang et al, 2023 (CDSS)^24^
	Hospital VTE prevention. AI-CDSS with automated data collection, scoring, and physician reminders	Retrospective clinical implementation study	19% reduction in VTE incidence	Zhou et al, 2021^25^
	Prediction of no clinical improvement after vein ablation in CEAP C2-C4 disease. XGBoost	33,924 patients with 1-year follow-up after vein ablation; lack of improvement defined as change in VCSS ≤ 0	Preoperative XGBoost AUROC = 0.94; intraoperative and postoperative models reached AUROC = 0.97; no independent external validation	Li et al, 2025 (Ablation)^26^
	Prediction of VTE within 30 days after endovenous thermal ablation. ML model	21,549 patients undergoing endovenous thermal ablation	XGBoost test-set AUC = 0.711; outcome incidence was 1.59% and validation was internal only	Tabari et al, 2025^27^
Imaging-based VTE detection	Ultrasound-image detection of features associated with increased VTE risk. Multisequence CNN/ResNetV2	201 patients contributing 801 femoral-vein ultrasound acquisitions	AUC = 0.74 for the soft-attention model (0.69 without attention); sensitivity = 0.73 and specificity = 0.68; no external validation	Huang et al, 2024 (Ultrasound)^28^
	Hemodynamic analysis of dynamic DSA sequences to stratify PE risk and guide thrombectomy. AI novel imaging model	168 patients undergoing venous intervention, with an additional nonoverlapping 50-patient historical pilot cohort	AUC = 0.88 in the reported test set (n = 60); treatment-effect estimates were retrospective and post hoc rather than randomized causal comparisons	Kang et al, 2026^29^
	CT-based thrombus-volume and embolic-burden analysis. AI plus dual-energy CT radiomics	136 patients with acute PE: 81 training and 55 testing	Test-set AUCs = 0.63 and 0.58 for two radiomics classification settings; no external validation	Gotta et al, 2024^30^
Bleeding management and complications	Warfarin dose prediction. Relevance vector machine and regression models	5052 patients from the International Warfarin Pharmacogenetics Consortium dataset	Primary dose-regression performance: RMSE 11.6 mg/wk and MAE 8.4 mg/wk; AUC was not applicable to the primary end point	Sharabiani et al, 2015^31^
	Anticoagulation adherence support. AI mobile application	Anticoagulated patients; AI app vs control	Adherence 90.5% vs 50% in controls, reported as a 67% improvement	Labovitz et al, 2017^32^
	Bleeding prediction in anticoagulated patients with cancer. ML model	1080 patients with cancer-associated thrombosis	Best mean AUROC = 0.65 ± 0.06 for random forest in the 1-90-day prediction experiment; no independent external validation	Grdinic et al, 2024^33^

*AI*, Artificial intelligence; *AI-CDSS*, artificial intelligence–based clinical decision support system; *AUC*, area under the curve; *AUROC*, area under the receiver operating characteristic curve; *CNN*, convolutional neural network; *DSA*, digital subtraction angiography; *DVT*, deep vein thrombosis; *EMR*, electronic medical record; *EVTA*, endovenous thermal ablation; *ICU*, intensive care unit; *ML*, machine learning; *NLP*, natural language processing; *NPV*, negative predictive value; *PE*, pulmonary embolism; *PPV*, positive predictive value; *PTS*, post-thrombotic syndrome; *VCSS*, Venous Clinical Severity Score; *VTE*, venous thromboembolism; *XGBoost*, extreme gradient boosting.

a In rare-outcome settings, high accuracy and specificity may simply reflect correctly classifying the large nonevent majority, making calibration and decision-curve–based clinical utility more informative than AUC alone.

### AI models for VTE risk prediction

Among predictive models, the most promising are those validated across large, diverse health systems or used for providing real-time perioperative decision support. Chen et al^11^ developed an EMR-based model using data from 159,001 patients and externally validated it in two independent cohorts totaling >500,000 patients in the United Kingdom and United States. The model had higher discrimination than the Padua score for VTE diagnosis and long-term risk prediction, supporting the transportability of discrimination across the evaluated cohorts, although its performance varied by the prediction target and population. MySurgeryRisk, developed with 52,117 surgical patients and validated in 22,300 patients, offers more than risk estimates. It was implemented via a mobile interface that displayed patient-specific risks, primary contributing factors, and surgeon outcome patterns (postoperative complications among individual surgeons compared to their colleagues over time).^13^ The workflow displayed individualized risk estimates and contributing factors that could support perioperative clinical review. Other pulmonary embolism (PE) machine learning models were trained using data from 42 to 200 hospitals.^14^, ^15^, ^16^ These models have higher discrimination than traditional risk scores, but only in selected cohorts. In our analysis, 35% (7/20) of the included studies had external validation, but 5 of 7 had high ROB, as determined using PROBAST+AI. Therefore, the most clinically credible models are not necessarily those with the highest reported discrimination, large sample size, or algorithmic complexity but are those that demonstrate reproducibility, prospective implementation, interpretability, and the ability to support a specific clinical decision.^11^^,^^13^, ^14^, ^15^, ^16^

### Detecting VTE in unstructured EMR data

Natural language processing (NLP) has been used to detect VTE cases that are often missed based on diagnosis codes, registries, or billing data. Verma et al^19^ showed that an NLP algorithm used in five Canadian hospitals identified VTE in hospitalized patients more accurately than International Classification of Diseases, 10th Revision (ICD-10) codes, supporting its role in surveillance, quality improvement, and retrospective outcome tracking. ClotCatcher similarly analyzed radiology reports to confirm VTE, and it was internally validated across two hospitals, illustrating how automated report interpretation could reduce the need for labor-intensive manual chart review.^20^ Woller et al^21^ also reported high sensitivity and specificity for NLP-based VTE identification within EMRs. These applications may support case identification, surveillance, and review of potentially missed diagnoses. Imaging-report NLP has also been used to identify PE, deep vein thrombosis, and clinically important incidental findings with high accuracy.^23^ Unlike general-purpose language models, these clinically focused systems are trained for specific tasks using institution data and outcomes. Their main immediate benefit is automated case detection and surveillance and not independent diagnosis. Their broader use requires validation across institutions due to differences in language, documentation, missing data, and medical record structure, which may affect portability.^19^, ^20^, ^21^^,^^34^

### Clinical decision support and intervention outcome prediction

AI can improve VTE outcomes through clinical decision-support systems (CDSS) combining automated risk assessment with clinician alerts. Huang et al^24^ evaluated an AI-based CDSS in a randomized trial involving 19,785 hospitalized patients. The system automatically integrated structured and unstructured EMR data; recalculated Caprini, Padua, bleeding-risk, and Wells scores every 6 hours or after new information was entered; and sent text-message alerts when patients were identified as high risk. The use of the system was associated with a 46% relative reduction in hospital-associated VTE (absolute risk reduction of 0.23), a 24% increase in mechanical prophylaxis, and a 9.7% increase in drug intensity compared with conventional prevention practices.^24^ These findings are clinically significant because, beyond risk prediction, they help bridge the gap between detecting risk and implementing prophylactic measures. Nevertheless, this study found no increase in the overall or appropriate prophylaxis rates due to variation in scattered clinical data across different data sources.^24^ Zhou et al^25^ reported a 19% lower VTE incidence following the implementation of an AI-CDSS in a retrospective study. Together, these studies suggest that the most effective AI systems may function as implementation tools that continuously reassess risk, identify missed prophylaxis opportunities, and deliver actionable recommendations at the point of care. AI has also been applied to venous intervention follow-up, including in the prediction of limited clinical improvement after vein ablation and VTE after endovenous thermal ablation.^26^^,^^27^ However, these procedural models require prospective testing before they can be considered comparable to hospital-based systems that have already demonstrated measurable reductions in VTE incidence.

### AI for imaging-based VTE detection

AI can help clinicians identify thromboembolic disease from ultrasound, computed tomography (CT), or angiographic data, although the evidence of clinical impact is less developed than that for CDSS. AidocBriefCase, a convolutional neural network, detected unreported events such as subsegmental emboli in cancer patients, which are possibly overlooked during routine interpretation.^22^ Such systems may support prioritization and secondary review. Kang et al^29^ described an application analyzing angiographic features (contrast flow, venous reflux, and filling defects) to classify PE risk. This approach demonstrated higher discrimination than the Wells score in the evaluated retrospective cohort, and it was associated with fewer PE cases and less severe post-thrombotic syndrome at 12 months.^29^ Lower-risk classifications were associated with less frequent thrombectomy; these retrospective, post hoc findings do not establish a treatment effect.^29^ Conversely, ultrasound and CT models show modest performance for VTE risk detection and complication prediction.^28^^,^^30^ These findings suggest that imaging tools may support case prioritization and clinician-reviewed intervention planning.

### AI tools for bleeding management and complications

AI tools applied in anticoagulation focus on medication selection, adherence, and bleeding prediction. Sharabiani et al^31^ showed that machine learning models had fewer dose-prediction errors than the evaluated traditional models. Labovitz et al^32^ reported that an AI-enabled app improved adherence from 50% to 90.5%, highlighting AI’s potential to modify behaviors linked to treatment effectiveness. Machine learning also estimated bleeding risk in cancer patients more accurately than CAT-BLEED, although the overall predictive performance remained moderate.^33^ The current evidence indicates that AI is likely more effective in enhancing adherence and assisting with medication management in the short term than in making autonomous decisions about starting or stopping anticoagulation without clinician oversight.

### Bias assessment

Of the 23 included studies, 20 prediction/detection studies were assessed using PROBAST+AI. Specifically, 90.0% (18/20) of the studies had a high overall ROB (Fig 1). The two low-ROB studies were those by Ren et al,^13^ which used prospective temporal validation, and Verma et al,^19^ which used a separate validation cohort. The analysis domain was high risk in 90.0% of studies due to overfitting, data-driven model selection, limited event counts, and reliance on internal validation alone. (Supplementary Table, online only). Meaningful external or prospective validation was conducted in 35.0% (7/20), but 71.4% remained at high ROB. Calibration was absent in 70.0% (14/20) of the studies.

**Fig 1:**
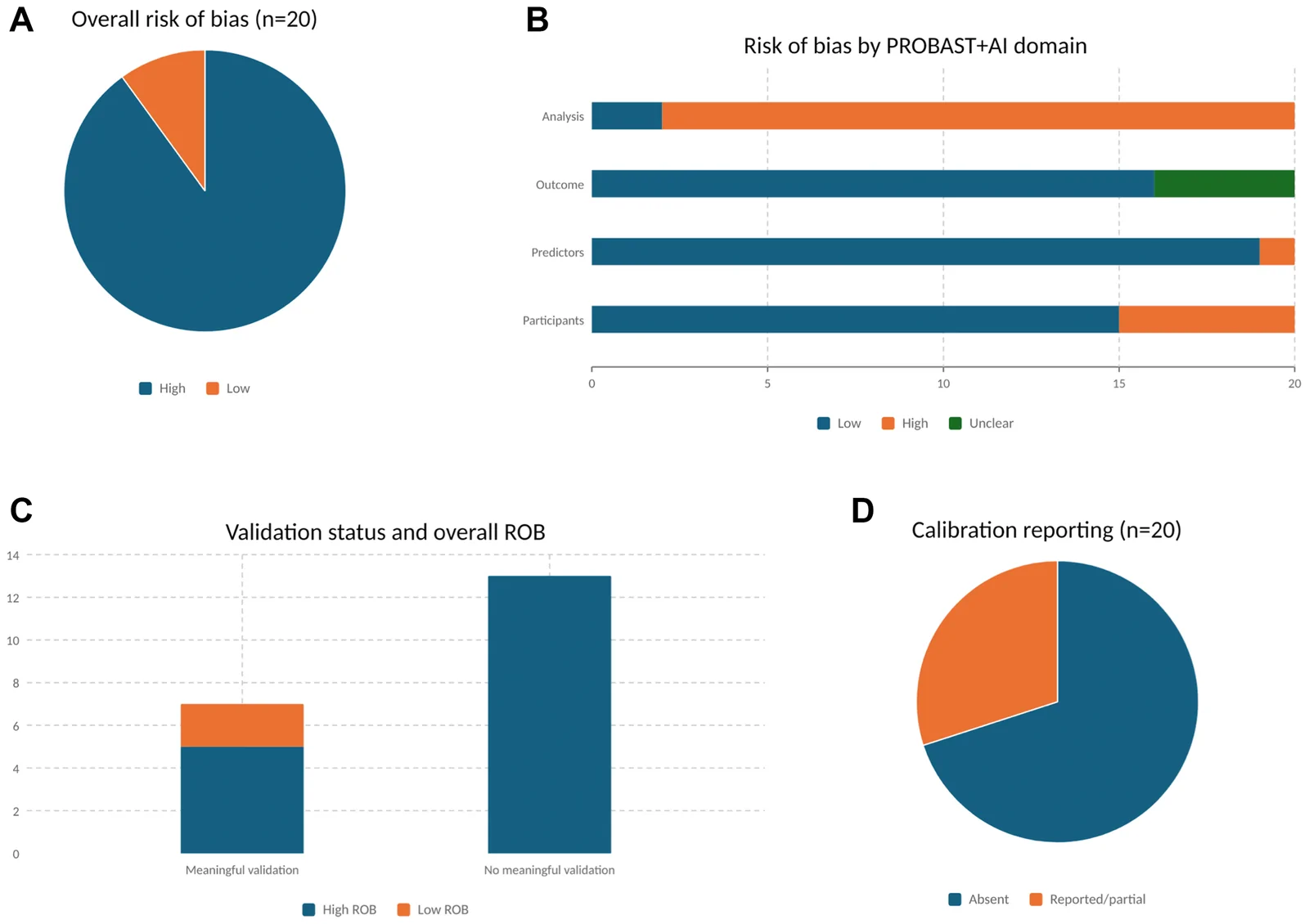
PROBAST+AI risk-of-bias assessment of AI studies in venous thromboembolism care. **A,** Overall risk-of-bias classification among the 20 studies assessed using PROBAST+AI: 18 studies (90.0%) were rated high risk, and two studies (10.0%) were rated low risk. **B,** Domain-level judgments showed high risk in 18 studies (90.0%) for analysis, 5 (25.0%) for participants, and 1 (5.0%) for predictors; the outcome domain was low risk in 16 studies (80.0%) and unclear in 4 (20.0%). **C,** Meaningful external or prospective validation was reported in 7 studies (35.0%); 5 of these 7 (71.4%) remained at high risk, whereas all 13 studies without meaningful validation were high risk. **D,** Calibration was absent in 14 studies (70.0%) and reported fully or partially in 6 studies (30.0%). AI, Artificial intelligence.

## Discussion

This review examines AI in VTE for improving diagnosis, risk prediction, and management. These models may support physicians and advanced practice providers if they are externally validated, integrated into clinical workflows, provide interpretable outputs, and demonstrate improved outcomes. Despite these advances, significant limitations persist, hindering broad clinical implementation. These limitations include data security, data quality,^34^ limited prospective external validation, population bias, and limited model interpretability. The “black box” nature of AI models can obscure how AI reaches decisions, reducing transparency in clinical decision-making and increasing the risk of errors.^35^ Post hoc explanation methods such as SHAP and LIME may improve the interpretability of individual model outputs, although they do not establish model validity or causality.^36^^,^^37^

The Food and Drug Administration has addressed predetermined change control plans for AI-enabled medical-device software and published Good Machine Learning Practice principles.^38^, ^39^, ^40^ Reporting and evaluation should follow stage-appropriate frameworks, including TRIPOD+AI, CONSORT-AI, SPIRIT-AI, CLAIM, and DECIDE-AI.^41^, ^42^, ^43^, ^44^, ^45^ Implementation frameworks additionally emphasize local validation, lifecycle monitoring, predefined uncertainty thresholds, auditability, clinician escalation pathways, and continued human oversight.^46^^,^^47^ The current VTE systems remain task-specific decision-support tools rather than autonomous agents.^48^ Agentic AI remains conceptual in VTE care, and any future workflow orchestration would require implementation safeguards, governance, and continued clinician control,^49^ as proposed in Fig 2.

**Fig 2:**
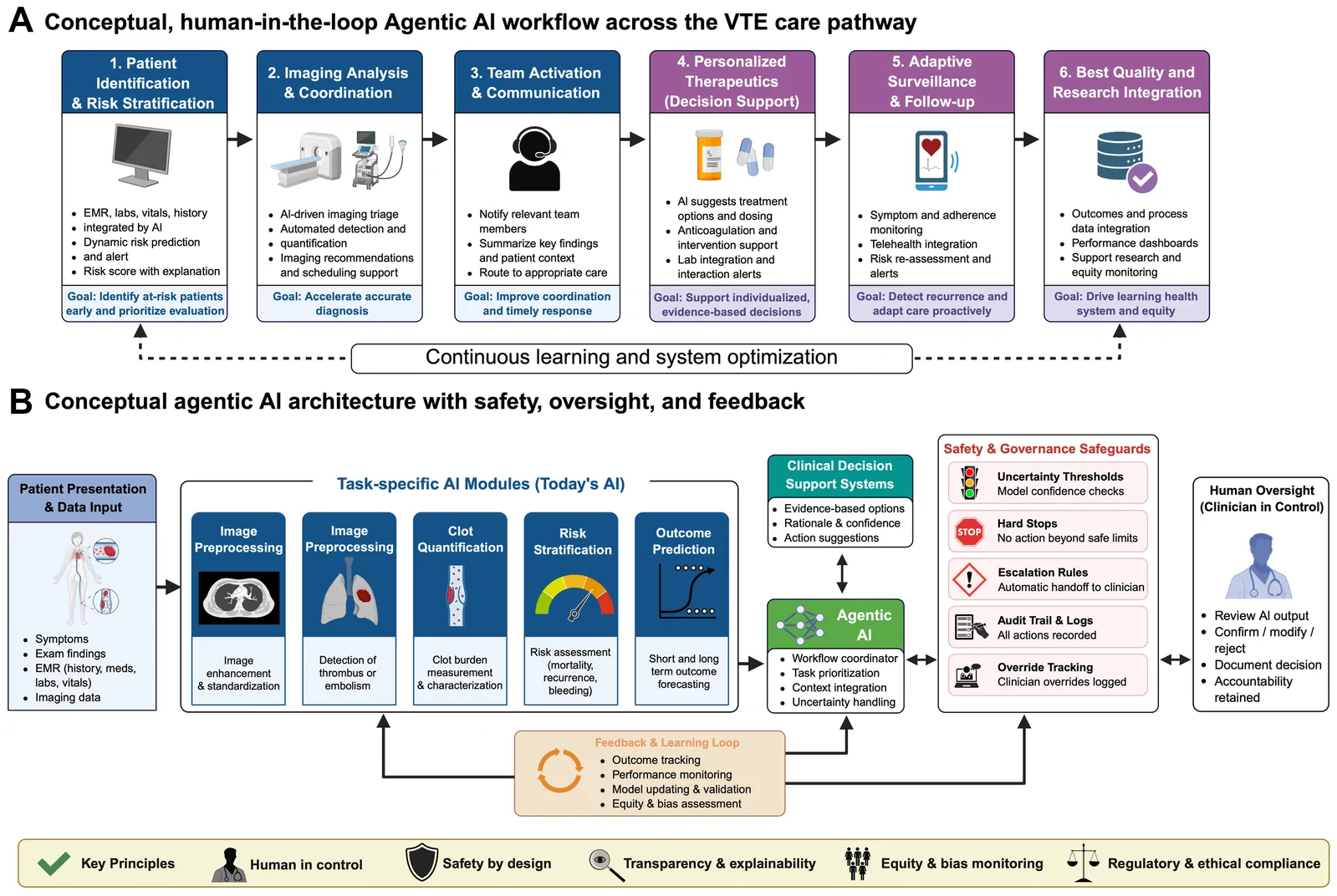
Conceptual, human-in-the-loop framework for future agentic AI in VTE care. **A,** Proposed workflow spanning patient identification, imaging coordination, multidisciplinary team activation, personalized treatment decision support, adaptive surveillance, and quality-improvement data integration. AI-generated recommendations and interim reviews remain subject to clinician confirmation rather than autonomous execution. **B,** Conceptual architecture in which task-specific modules perform image preprocessing, VTE detection, clot quantification, risk stratification, and outcome prediction under coordination by an orchestrator. Clinical review, uncertainty thresholds, hard stops, escalation rules, bias monitoring, audit logs, override documentation, and outcome tracking provide safeguards. This framework is investigational and requires prospective clinical, regulatory, and implementation evaluation before deployment. Created in BioRender. Moreno, O. (2026) https://BioRender.com/cs1igcn. *AI*, Artificial intelligence; *EMR*, electronic medical record; *VTE,* venous thromboembolism.

## Conclusions

AI has substantial potential to improve VTE prediction, detection, and management, but most existing studies have a high ROB. Agentic AI remains investigational. Future research should continue to assess prospective testing, transparent reporting, ongoing monitoring, and human oversight before endorsing or implementing AI systems in clinical settings.

## Funding

This work was supported by the 10.13039/100008877Conrad and Caroline Jobst Foundation Fellowship awarded to O.M.

## Disclosures

None.
