# Supplementary material for: Integrating artificial intelligence into venous thromboembolism care: Predictive models, implementation challenges, and future directions
Source: J Vasc Surg Venous Lymphat Disord. 2026 Aug 31;15(1):102608. doi: 10.1016/j.jvsv.2026.102608 (PMC13634517; doi:10.1016/j.jvsv.2026.102608)
Supplement: Supplementary file 1 [file mmc1.docx]

| **Study** | **AI_Approach** | **Clinical_Domain** | **Design** | **Study_Type** | **PROBAST_Applicable** | **Appropriate_Tool** | **Sample_Size** | **D1_Participants** | **D1_Rationale** | **D2_Predictors** | **D2_Rationale** | **D3_Outcome** | **D3_Rationale** | **D4_Analysis** | **D4_Rationale** | **Overall_ROB** | **Overall_Rationale** | **External_Validation** | **Calibration** | **AUC_Internal** | **AUC_External** | **Key_Limitation** |
| --- | --- | --- | --- | --- | --- | --- | --- | --- | --- | --- | --- | --- | --- | --- | --- | --- | --- | --- | --- | --- | --- | --- |
| Chen et al. 2024 | LightGBM gradient boosting | VTE diagnosis and 1-year risk | Retrospective multicohort EHR study | PREDICTION | Yes | PROBAST+AI | 159,001 patients (development cohort); 683,763 patients across all cohorts | **LOW** | Large, diverse development cohort with explicit eligibility; retrospective EHR selection and completeness exclusions noted. | **LOW** | Predictors were routinely available structured EHR variables and time windows were defined before the index date. | **LOW** | VTE defined from diagnosis codes/concepts; exclusions and cutoff dates were prespecified, though outcome misclassification remains possible. | **HIGH** | Nested CV and two external datasets were used, but cohort-specific imputation, data-driven feature screening, and limited calibration reporting create analysis concerns. | **HIGH** | Analysis-domain concerns despite large external testing. | Yes - UK Biobank and All of Us | Partial - Brier score reported; no calibration slope/intercept | 0.83 (large diagnosis model, MSDW holdout) | 0.80 (UK Biobank); 0.82 (All of Us), large diagnosis model | Retrospective coded outcomes; external performance varied by cohort and prediction target. |
| Wang et al. 2020 | Random forest and other ML models | Hospital VTE risk | Retrospective derivation with consecutive clinical validation | PREDICTION | Yes | PROBAST+AI | 1,955 patients (376 derivation; 1,579 validation) | **HIGH** | Small, artificially balanced derivation sample (188 VTE/188 non-VTE) is not representative of clinical prevalence. | **LOW** | Clinical predictors were available before outcome assessment. | **LOW** | VTE case status was clinically defined, but details of blinded outcome ascertainment were limited. | **HIGH** | Small sample, many candidate models, class balancing, and limited calibration/overfitting assessment. | **HIGH** | Nonrepresentative sampling and high analysis risk. | Yes - single-center consecutive clinical validation | No | 0.856 ± 0.049 (random forest, repeated internal test sets) | 0.782 (random forest, consecutive clinical validation cohort) | Balanced case-control derivation and small sample limit transportability. |
| Ren et al. 2022 (MySurgeryRisk) | Random forest and generalized additive models | Postoperative complications including VTE | Retrospective development; prospective temporal validation | PREDICTION | Yes | PROBAST+AI | 58,236 patients (74,417 procedures) | **LOW** | All adult inpatient surgical procedures were included with clear temporal cohorts. | **LOW** | Automated EHR predictors were available in real time before postoperative outcomes. | **LOW** | Postoperative complications were prospectively captured using predefined definitions. | **LOW** | Large development set, prospective validation (22,300 procedures), and prespecified performance evaluation; remaining concern is single-system transportability. | **LOW** | Prospective temporal validation supports low overall risk. | Yes - prospective temporal validation | Partial | Not reported separately for VTE in the extracted article results | Not reported separately for VTE in the prospective validation results | Single health system; multi-outcome model rather than a VTE-specific model. |
| Shen et al. 2022 | XGBoost | 10-day inpatient PE prediction | Retrospective multicenter cohort | PREDICTION | Yes | PROBAST+AI | 1,991,983 admissions | **LOW** | Very large dataset spanning 44 US institutions with clear adult admission criteria. | **LOW** | First-3-hour EHR predictors precede PE occurrence within 10 days. | **LOW** | PE outcome was defined from hospital data; coding/ascertainment details may allow some misclassification. | **HIGH** | Holdout testing and 32-site external validation were strong, but retrospective design, limited calibration assessment, and potential site/coding heterogeneity remain. | **HIGH** | External discrimination was excellent, but calibration and retrospective outcome ascertainment were insufficiently assessed. | Yes - 32 external institutions | No | 0.87 (20% holdout from training-site data) | Mean 0.88 across 32 external institutions; range 0.79-0.93 | Calibration was not reported; retrospective outcome ascertainment across heterogeneous sites. |
| Guan et al. 2023 | Random forest, XGBoost, SVM | ICU VTE prediction | Retrospective eICU cohort | PREDICTION | Yes | PROBAST+AI | 109,044 patients | **LOW** | Large multicenter ICU database; inclusion criteria and train/validation split reported. | **LOW** | Clinical and laboratory predictors were available during ICU care before VTE occurrence. | **UNCLEAR** | VTE identification relied on database coding; independent/blinded adjudication was not described. | **HIGH** | Stepwise selection plus random split validation, severe class imbalance, and no calibration analysis increase overfitting/optimism risk. | **HIGH** | No independent external validation and high analysis risk. | No - random split only | No | 0.9492 (XGBoost, highest reported validation AUC); RF 0.9378 | Not reported; validation was an internal split | Rare outcome, severe class imbalance, and no independent external validation. |
| Danilatou et al. 2022 | AutoML/XGBoost and multiple ML models | ICU mortality in VTE and cancer | Retrospective database development and external validation | PREDICTION | Yes | PROBAST+AI | 2,468 patients (development); 2,659 patients (external validation) | **LOW** | MIMIC-III VTE cohort was clearly selected; eICU supplied a separate external VTE cohort. | **LOW** | Routine ICU clinical/laboratory predictors were measured before mortality outcomes. | **LOW** | Mortality is objective and reliably captured. | **HIGH** | Extensive model/feature comparisons, missing-data handling concerns, and limited calibration reporting create optimism risk. | **HIGH** | External validation does not offset analysis-domain limitations. | Yes - eICU (4,385 VTE patients) | No | 0.93 (early mortality in VTE, MIMIC-III) | 0.87 (early mortality in VTE, eICU) | Retrospective databases, extensive model search, and limited calibration; outcome was mortality rather than VTE occurrence. |
| Jin et al. 2022 | LDA, logistic regression, tree, RF, SVM | Cancer-associated DVT prediction | Retrospective single-center cohort | PREDICTION | Yes | PROBAST+AI | 1,035 patients | **LOW** | Eligible cancer patients undergoing Doppler ultrasonography were clearly described, but verification/selection bias is possible. | **LOW** | Candidate predictors were clinically available. | **LOW** | DVT was determined by Doppler ultrasonography. | **HIGH** | LASSO selection, multiple model comparisons, single random split, and modest event count increase overfitting risk despite Brier/calibration curves. | **HIGH** | Analysis and selection-bias concerns; no external validation. | No | Yes - Brier score and calibration curve | 0.773 (LDA with D-dimer; LR 0.772, 30% test set) | Not reported | Only patients undergoing ultrasonography were included; single-center split-sample design. |
| Nafee et al. 2020 | Super learner ensemble | VTE prediction in acutely ill medical patients | Secondary analysis of randomized trial data | PREDICTION | Yes | PROBAST+AI | 6,459 patients analyzed (7,513 enrolled) | **LOW** | Well-characterized APEX trial cohort, but highly selected and not broadly representative. | **LOW** | Baseline predictors were measured before VTE outcomes. | **LOW** | Trial outcomes were prospectively adjudicated. | **HIGH** | Model developed/evaluated in one trial cohort without independent validation; many candidate learners and composite including asymptomatic DVT. | **HIGH** | Selected trial population and no external validation. | No | Yes - observed vs predicted calibration reported | 0.69 cross-validated c-statistic (ML); 0.68 reduced ML | Not reported | Selected elderly clinical-trial population; composite endpoint included asymptomatic DVT. |
| Verma et al. 2022 | Rule-based NLP algorithms | VTE detection from radiology reports | Cross-sectional retrospective validation study | DETECTION | Yes | PROBAST+AI | 10,049 admissions (6,049 development; 4,000 validation) | **LOW** | Five hospitals and separate training (1,551) and validation (4,000 hospitalizations) samples. | **LOW** | Radiology text was available before NLP classification; algorithm rules were developed in a separate sample. | **LOW** | Manual review served as the reference standard. | **LOW** | Large independent validation sample with direct accuracy estimates; calibration is not relevant for largely rule-based classification outputs. | **LOW** | Clear separate validation against manual review. | Yes - separate 4,000-hospitalization validation sample | Not applicable/not reported | Algorithm-specific: e.g., CT-Acute PE train AUC 0.96 | 0.96 for CT-All PE and 0.96 for US-DVT in the main validation/test reporting | Performance may depend on local radiology-report language and workflow. |
| Wang et al. 2023 (ClotCatcher) | BioBERT NLP | VTE adjudication from radiology reports | Retrospective two-hospital development/validation | DETECTION | Yes | PROBAST+AI | 2,273 imaging studies | **LOW** | Two hospital datasets were explicitly sampled. | **LOW** | Input radiology reports were distinct from physician labels used as reference. | **LOW** | Clinician adjudication of radiology studies was the reference standard. | **HIGH** | Random splits, extensive model/paraphrasing comparisons, and training on combined-site data mean reported site tests are not fully external. | **HIGH** | Validation was internal to the two source hospitals and calibration was not reported. | No - site holdouts were derived from source data | No | Internal/site-holdout results varied by training configuration | 0.980 on EUH and 0.995 on GMH for the best reported configurations | Small labeled sample and potential optimism from multiple model configurations. |
| Woller et al. 2021 | NLP EMR interrogation tool | VTE detection from imaging reports | Retrospective diagnostic validation | DETECTION | Yes | PROBAST+AI | 400 charts | **HIGH** | Case-enriched sampling selected 100 positive and 100 negative studies for each of PE and DVT rather than a representative clinical series. | **LOW** | NLP interpreted imaging reports independently of manual chart review. | **LOW** | Manual chart review was the reference standard. | **HIGH** | Case-control style sampling prevents prevalence-dependent performance assessment; no external validation or calibration. | **HIGH** | Nonrepresentative sampling and limited sample size. | No | No | AUC not reported; sensitivity 93.3%, specificity 99.6% overall | Not reported | Artificially balanced sample; prevalence-dependent performance may not generalize. |
| Wiklund et al. 2023 | Commercial AI algorithm for CT PE detection | Incidental PE detection in cancer CT | Retrospective single-center diagnostic cohort | DETECTION | Yes | PROBAST+AI | 1,069 patients (1,892 CT studies) | **LOW** | Consecutive elective chest CT cohort with 1,069 patients/1,892 studies. | **LOW** | AI processed CT images without access to manual reread labels. | **LOW** | All reports and images were rereviewed to establish incidental PE. | **HIGH** | Only 75 positive studies, no model recalibration, and single-center retrospective evaluation; AUC/calibration not reported. | **HIGH** | Small number of positives and single-center retrospective evaluation. | Yes - independent evaluation of a pre-existing AI algorithm | No | AUC not reported; sensitivity 90.7%, specificity 99.8% | Not reported | Only 75 incidental-PE-positive studies; false negatives clustered in small clot burden. |
| Pham et al. 2014 | NLP plus supervised ML | PE/DVT detection from French radiology reports | Retrospective corpus study | DETECTION | Yes | PROBAST+AI | 573 radiology reports | **HIGH** | Single-center corpus and one physician's annotation limit representativeness. | **LOW** | Text-derived concepts were used as predictors. | **UNCLEAR** | One physician annotated the corpus; blinding and inter-rater reliability were not established. | **HIGH** | Small corpus, feature engineering/model selection on the same dataset, and no independent external validation. | **HIGH** | Small single-center corpus and uncertain reference standard reproducibility. | No | No | AUC not reported; F1 approximately 0.98 for PE and 1.00 for DVT | Not reported | Single annotator and no independent external validation. |
| Huang et al. 2024 | Multisequence attention CNN | Spontaneous echo contrast detection on femoral-vein ultrasound | Retrospective imaging study | DETECTION | Yes | PROBAST+AI | 201 patients (801 ultrasound acquisitions) | **HIGH** | Only 201 patients (801 acquisitions) from archival single-center imaging. | **LOW** | Ultrasound sequences were model inputs. | **UNCLEAR** | Expert SEC labeling was used, but blinding/reproducibility details were limited. | **HIGH** | 80/20 split, small patient count, multiple acquisitions per patient, and no external validation raise leakage/overfitting concerns. | **HIGH** | Small single-center split-sample study. | No | No | 0.74 (soft-attention SEC model; 0.69 without attention) | Not reported | Small patient cohort with repeated acquisitions and possible patient-level correlation. |
| Li et al. 2025 | XGBoost and five comparator ML models | Lack of clinical improvement after varicose-vein ablation | Retrospective registry cohort | PREDICTION | Yes | PROBAST+AI | 33,924 patients | **LOW** | Large VQI registry cohort with explicit procedure and disease criteria. | **LOW** | Preoperative predictors precede 1-year outcome; intra/postoperative models are separate use cases. | **LOW** | Outcome was defined from change in VCSS at 1 year. | **HIGH** | Large predictor set (226), multiple models/stages, complete-follow-up selection, and random split without external validation create optimism/selection concerns. | **HIGH** | No external validation and substantial feature/model search. | No | Yes - Brier scores reported | 0.94 preoperative XGBoost; 0.97 intraoperative and postoperative models | Not reported | Complete 1-year follow-up requirement and no geographic or temporal external validation. |
| Tabari et al. 2025 | CART, optimal trees, RF, XGBoost | 30-day DVT after endovenous thermal ablation | Retrospective NSQIP cohort | PREDICTION | Yes | PROBAST+AI | 21,549 patients | **LOW** | Large national surgical database with explicit procedure criteria. | **LOW** | Demographics, comorbidities and labs were preoperative. | **LOW** | 30-day postoperative DVT is a predefined NSQIP outcome. | **HIGH** | Rare outcome (1.59%), holdout-only validation, broad candidate-variable search, and no calibration/external validation. | **HIGH** | Rare events and internal holdout only. | No | No | 0.711 XGBoost test-set AUC; train AUC 1.00 | Not reported | Low event rate and internal holdout validation only. |
| Kang et al. 2026 | Transformer-UNet plus LSTM radiomics | PE risk from dynamic DSA in DVT | Retrospective single-center cohort | PREDICTION | Yes | PROBAST+AI | 168 patients (plus 50-patient pilot cohort) | **HIGH** | Small single-center cohort; a separate historical pilot informed target effect/sample size. | **HIGH** | Intraprocedural image features may be influenced by treatment/procedure and were derived using complex transfer learning. | **LOW** | Subsequent PE was clinically assessed, though details of blinded adjudication are limited. | **HIGH** | Very small sample for deep learning, post hoc simulation of treatment strategy, and no true independent validation. | **HIGH** | Severe overfitting and confounding risk. | No | No | 0.88 on the reported independent test set (n=60) | Not reported | Small retrospective cohort; estimated clinical effects were post hoc rather than causal. |
| Gotta et al. 2024 | Gradient-boosted radiomics classifier | Complicated course in acute PE | Retrospective single-center imaging cohort | PREDICTION | Yes | PROBAST+AI | 136 patients | **HIGH** | Small retrospective cohort and outcome-group selection. | **LOW** | DECT radiomic features were measured at baseline. | **UNCLEAR** | Composite 'complicated course' included hospitalization/IMC and other events; outcome definition may be influenced by clinician decisions. | **HIGH** | Very small sample, 107 candidate radiomic features, stepwise reduction, and train/test split without external validation. | **HIGH** | Small sample and data-driven radiomics pipeline. | No | No | 0.63 and 0.58 for two radiomics classification settings in the test dataset | Not reported | Small sample, high-dimensional radiomics, and no external validation. |
| Sharabiani et al. 2015 | RVM classification plus regression | Warfarin dose prediction | Retrospective secondary analysis of IWPC data | PREDICTION | Yes | PROBAST+AI | 5,052 patients | **LOW** | Large international pharmacogenetic dataset, though exclusions/missingness reduce the final analytic sample. | **LOW** | Clinical/genetic predictors are available before stable dose estimation. | **LOW** | Stable therapeutic warfarin dose is an objective continuous outcome. | **HIGH** | Two-stage modeling, repeated feature/model comparisons, and no independent external validation; performance reported mainly as RMSE/MAE rather than calibration. | **HIGH** | No external validation and complex data-driven model selection. | No | No | AUC not applicable to the primary dose-regression endpoint; RMSE 11.6 and MAE 8.4 | Not reported | Primary endpoint was warfarin-dose regression rather than a VTE outcome. |
| Grdinic et al. 2024 | Lasso/Ridge, random forest, XGBoost | Bleeding in cancer-associated thrombosis | Retrospective registry cohort | PREDICTION | Yes | PROBAST+AI | 1,080 patients | **LOW** | Consecutive registry-based CAT cohort with predefined follow-up windows. | **LOW** | Clinical/biochemical predictors were collected before each bleeding prediction window. | **LOW** | Major bleeding and clinically relevant nonmajor bleeding used standard definitions. | **HIGH** | 488 candidate attributes, repeated cross-validation/model selection, limited event counts, and no external validation/calibration. | **HIGH** | High-dimensional analysis with limited events and no external validation. | No | No | Best mean AUROC 0.65 ± 0.06 (random forest, 1-90 day experiment); other horizons/models ~0.59-0.64 | Not reported | Moderate discrimination with no independent external validation. |
